# Application of the CRISPR/Cas9 system for modification of flower color in *Torenia fournieri*

**DOI:** 10.1186/s12870-018-1539-3

**Published:** 2018-12-05

**Authors:** Masahiro Nishihara, Atsumi Higuchi, Aiko Watanabe, Keisuke Tasaki

**Affiliations:** 1 Iwate Biotechnology Research Center, 22-174-4, Narita, Kitakami, Iwate, 024-0003 Japan; 2 Present Address: Tokyo University of Agriculture, Atsugi, Kanagawa 243-0034 Japan

**Keywords:** CRISPR/Cas9, Flavanone 3-hydroxylase, Flower color, Genome editing, *Torenia fournieri*

## Abstract

**Background:**

CRISPR/Cas9 technology is one of the most powerful and useful tools for genome editing in various living organisms. In higher plants, the system has been widely exploited not only for basic research, such as gene functional analysis, but also for applied research such as crop breeding. Although the CRISPR/Cas9 system has been used to induce mutations in genes involved in various plant developmental processes, few studies have been performed to modify the color of ornamental flowers. We therefore attempted to use this system to modify flower color in the model plant torenia (*Torenia fournieri* L.).

**Results:**

We attempted to induce mutations in the torenia flavanone 3-hydroxylase *(F3H*) gene, which encodes a key enzyme involved in flavonoid biosynthesis. Application of the CRISPR/Cas9 system successfully generated pale blue (almost white) flowers at a high frequency (ca. 80% of regenerated lines) in transgenic torenia T_0_ plants. Sequence analysis of PCR amplicons by Sanger and next-generation sequencing revealed the occurrence of mutations such as base substitutions and insertions/deletions in the *F3H* target sequence, thus indicating that the obtained phenotype was induced by the targeted mutagenesis of the endogenous *F3H* gene.

**Conclusions:**

These results clearly demonstrate that flower color modification by genome editing with the CRISPR/Cas9 system is easily and efficiently achievable. Our findings further indicate that this system may be useful for future research on flower pigmentation and/or functional analyses of additional genes in torenia.

**Electronic supplementary material:**

The online version of this article (10.1186/s12870-018-1539-3) contains supplementary material, which is available to authorized users.

## Background

Flower color is one of the most important traits in ornamental flowers, and plant breeders have devoted a great deal of effort to the production of varieties with new flower colors. Although these varieties have mainly been generated by traditional cross-breeding or tissue culture techniques such as embryo rescue to produce interspecific hybrids, several have also been produced using modern biotechnology methods, such as artificial mutagenesis by exposure to UV, ionizing radiation or chemical mutagens, and genetic transformation. For example, the production of flower-color mutants has been reported in various ornamental flowers, including cyclamens, Catharanthus and chrysanthemum, by ion-beam irradiation [1, 2]. We also recently induced a flower color change from blue to pink in Japanese gentian, with a frequency as high as 8.3%, by ion-beam irradiation [3]. The most problematic aspect of this technique, however, is that flower color cannot be predicted in advance, and which genes are mutated remains unknown in mutagenesis breeding. In particular, mutations in most cases are randomly induced, with several genes simultaneously mutated in various ways, such as by insertion/deletions (In/Dels), base substitutions and chromosome rearrangements, thereby hindering the effective acquisition of a desirable flower color. If desirable flower-color mutants are obtained, further screening of elite lines exhibiting no defects in other traits is required. Genetic transformation, in contrast, is the most straightforward approach to produce desired flower colors without changing other traits. In fact, blue-hued carnations and roses produced by genetic transformation have been commercialized by Suntory Ltd. for many years [4]. Similarly, a transgenic approach can be applied to improve various flower traits, including flower pigmentation, fragrance and shape, flowering time and disease resistance [5].

Flower color modification by genetic transformation has been attempted for several decades [6, 7]. In early years, white-colored flowers generated by antisense or RNAi targeting of structural genes, e.g. the chalcone synthase (*CHS*) gene, were produced in several plant species such as petunia, tobacco and chrysanthemum. Transcription factors such as *MYB* and *bHLH* have also been used as target genes for genetic engineering [8, 9]. Flower color mutants with deficiencies in certain genes are especially valuable to produce desirable flower colors in genetic engineering experiments. For example, one early study producing brick-red flowers in petunia used a triple mutant as a transformed host [10]. Blue-hued carnations or roses can also be produced by eliminating competition from the endogenous enzymes flavonoid 3*′*-hydroxylase (F3*′*H), dihydroflavonol 4-reductase (DFR) and flavonol synthase (FLS); consequently, downregulation of these genes in addition to the introduction of the flavonoid 3′5′-hydroxylase (*F3′5′H*) gene is necessary to accomplish the accumulation of high amounts of delphinidin-type anthocyanins [7]. Similarly, mutant lines are useful as breeding materials for the genetic engineering of interesting flower colors, but few materials are available in most floricultural species. Even if useful mutants exist, the incorporation of the mutant traits by traditional cross-breeding is time-consuming and arduous.

Many recent studies have focused on genome editing in higher plants [11–13]. One of the most powerful and reliable genome-editing methods is the CRISPR/Cas9-based system developed using the bacterial immune system. Flower color modification by CRISPR/Cas9-mediated mutagenesis of the *DFR*-*B* gene was recently achieved in Japanese morning glory [14]. This technique can undoubtedly be adapted not only for basic studies but also for applied studies such as crop breeding. The efficiency of this system in various plant species is currently being optimized; these efforts include modification of *Streptococcus pyogenes* SpCas9 nuclease, application of new variants of the genome modifying system from other bacterial species and redesign of single-guide RNA [15]. Furthermore, a DNA-free genome editing system with preassembled CRISPR-Cas9 ribonucleoproteins has been developed that may overcome GMO restrictions in plants [16]. New methods such as RNA-targeted genome editing and base editing by engineered deaminase have been developed more recently [17–19].

In this study, we attempted to modify flower colors by genome editing of a flavonoid biosynthetic-related gene using the basic CRISPR/Cas9 system. For this purpose, we used *Torenia fournieri*, a model plant widely applied for flower research [20]. We targeted the flavanone 3-hydroxylase (*F3H*) gene, a key enzyme gene in the flavonoid biosynthetic pathway (Additional file 1: Figure S1), because we had previously determined that an *F3H* mutation was responsible for the white flower color of a torenia cultivar [21]. *F3H* has also more recently been successfully edited using the CRISPR/Cas9 system in carrot calli [22], but its effect has not yet been evaluated in flowers. The results of the study reported here clearly demonstrate that the CRISPR/Cas9 system can be efficiently applied to modify flower color in torenia. Finally, we have also evaluated the efficiency and usefulness of genome editing for flower research.

## Results

### Flower pigmentation phenotypes of transgenic torenia plants

**Fig. 1:**
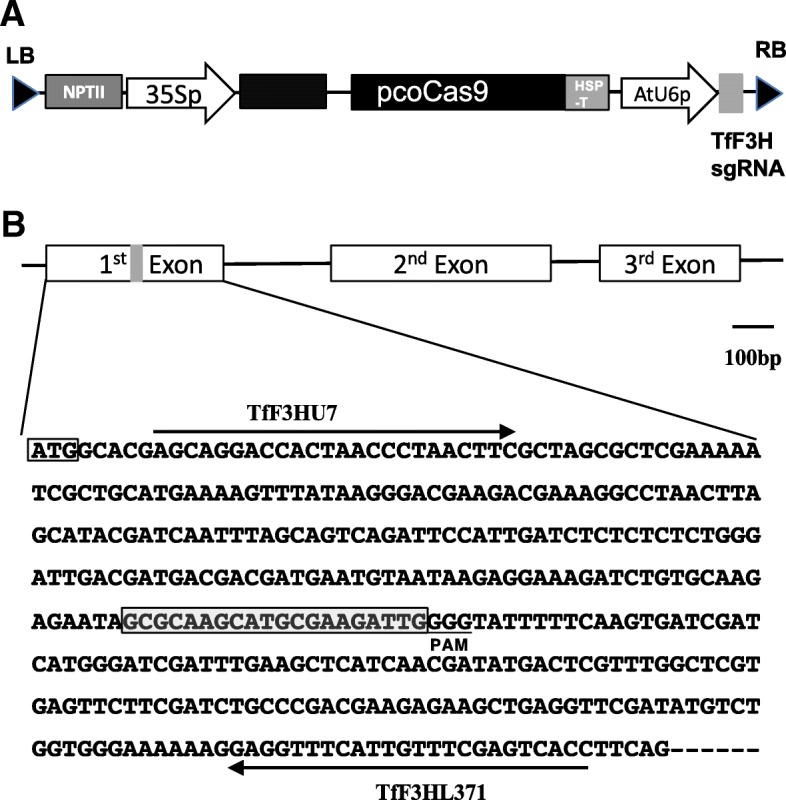
Schematic diagram of the binary vector and the torenia target sequence in the torenia *F3H* gene. **a** Schematic diagram of the T-DNA region of pSKAN-pcoCas9-TfF3H used in this study. NPTII, expression cassette of NOSp-nptII-AtrbcsTer; 35Sp, CaMV35S promoter; pcoCas9, plant codon-optimized Cas9 [35]; HSPter, Arabidopsis heat shock protein 18.2 terminator [36]; RB, right border; LB, left border; AtU6p, Arabidopsis small RNA U6–26 promoter; TfF3HsgRNA, torenia *F3H* targeted single-guide RNA. pcoCas9 contains an intron derived from the intervening sequence 2 *(IV2*) of the potato *St-LS1* gene [35]. **b** Genomic structure of the torenia *F3H* gene and exon 1 sequence. Boxes indicate exons, and lines between boxes indicate introns. Framed ATG indicates the start codon, and the gray box indicates the target site *F3H* sequence. The protospacer-adjacent motif (PAM) is underlined. Primers used for PCR amplification are also shown

**Fig. 2:**
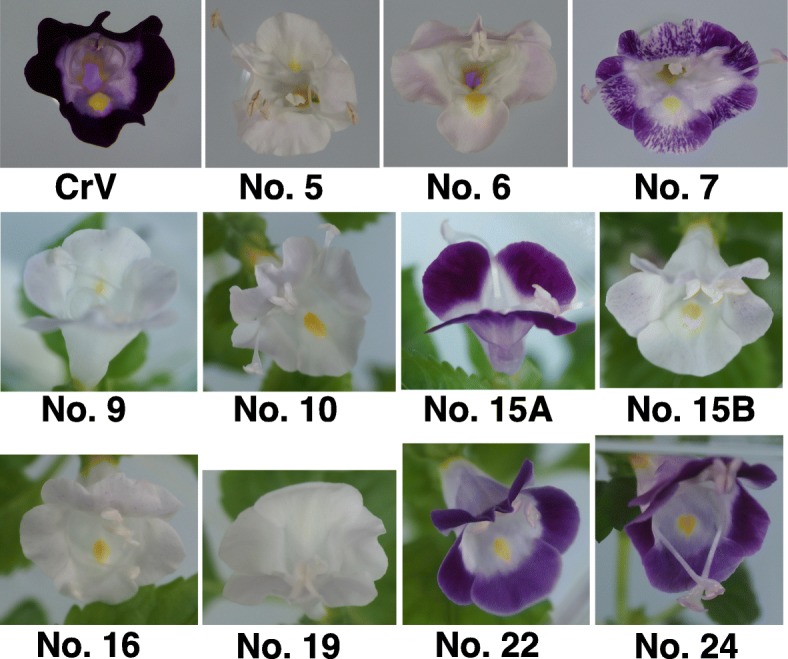
In vitro flowering phenotypes of transgenic torenia plants. Photographs were taken 3 to 8 months after inoculation with *Agrobacterium.* Numbers indicate transgenic plant lines. Line no. 15 had different-colored flowers and was divided into 15A and 15B

We constructed a binary vector using a plant codon-optimized *S. pyogenes* strain (pcoCas9) and a guide RNA-targeted exon 1 of the torenia *F3H* gene (Fig. 1) and then produced 24 transgenic torenia plants by *Agrobacterium*-mediated transformation. After several subcultures, the transgenic plants set flowers in vitro within 8 months. The earliest blossoms were observed less than 4 months after *Agrobacterium* infection. Representative flowers are shown in Fig. 2. The plants, which exhibited several different flower colors, are summarized in Table 1. In particular, 4 of the 24 lines had violet flowers, the same as wild-type untransformed control plants. Fifteen lines produced faint blue (almost white) flowers, while three lines (nos. 22–24) bore pale violet flowers, and line no. 15 set violet or faint blue flowers depending on the propagated stems. Interestingly, line no. 7 produced variegated flowers, but the phenotype was unstable. In several of the faint blue flower-colored lines, a few blue spots were observed (e.g. lines no. 9, 15B and 16 in Fig. 1; Table 1).

**Table 1:**
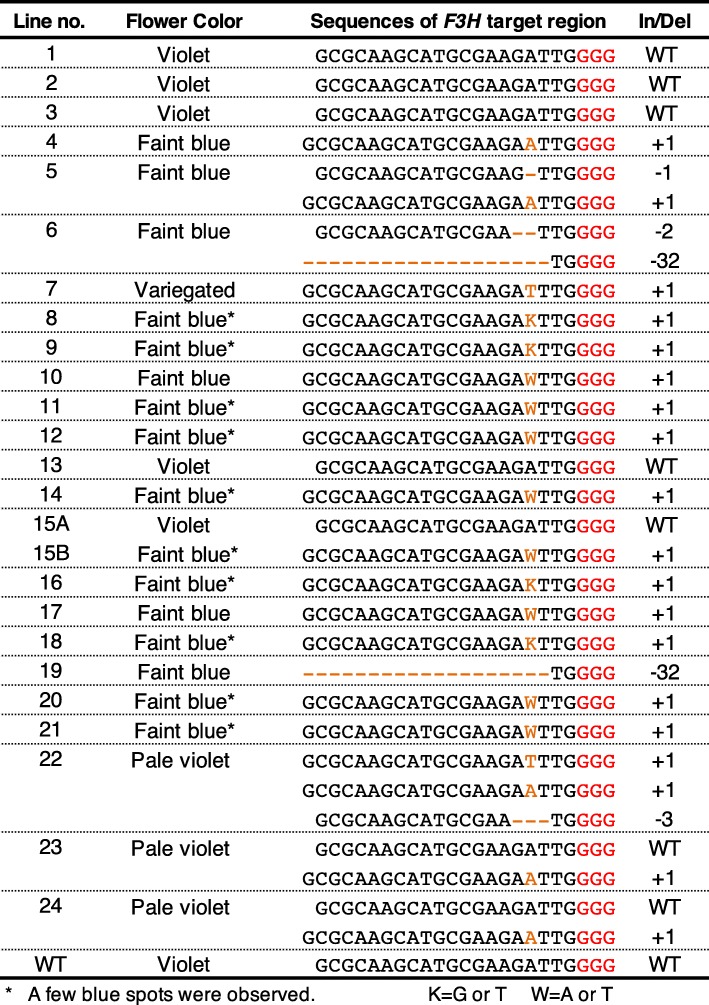
Flower color phenotypes and *F3H* target sequences determined by Sanger sequencing analysis

### Sequence analyses of the *F3H* target region

First, fragments amplified by PCR using primers TfF3HU7 and TfF3HL371 were directly subjected to sequencing analysis. Typical results are shown in Additional file 1: Figure S2. The sequence chromatogram of an untransformed WT plant contained a clear sequencing peak corresponding to the wild-type *F3H* sequence, whereas most transgenic plants showed sequence changes, such as single-base insertions, deletions or substitutions. Some lines, such as nos. 5, 6 and 23, had mixed-sequence chromatograms that were probably due to the presence of multiple edited sequences. Subcloning was also performed in some lines to confirm the sequences. According to the sequencing results, summarized in Table 1, all flower-color phenotypes coincided with the presence of mutated sequences of the *F3H* target region. More than 60% of transgenic lines (15/24) had flower color changes, from violet to faint blue (almost white). The faint blue flowers had mutations in both *F3H* alleles. Pale violet-flowered lines no. 23 and 24 contained two sequences: WT and + 1A alleles. The other pale violet line, no. 22, contained three sequences corresponding to -3 bp, + 1 T and + 1A alleles. In the case of torenia, single-base insertions were abundantly observed in edited sequences.

Because Sanger sequencing clearly revealed the results of genome editing in the target *F3H* region, we next performed a sequencing analysis on an Illumina Mi-seq next-generation sequencer to more efficiently obtain sequence information. Nine transgenic genome-edited torenia lines and the WT were selected and subjected to next-generation amplicon sequencing of the same *F3H* target region. The resulting data are summarized in Table 2 and Additional file 2. Sequence reads other than major reads were observed in all samples including the wild type; these reads represented less than 0.5% of total reads and were considered to be derived from PCR or NGS errors. The results of Sanger sequencing and NGS were basically consistent except for those of line no. 19. In line no. 19, a -2 bp edited sequence was also detected in addition to -32 bp editing determined by Sanger sequencing.

**Table 2:**
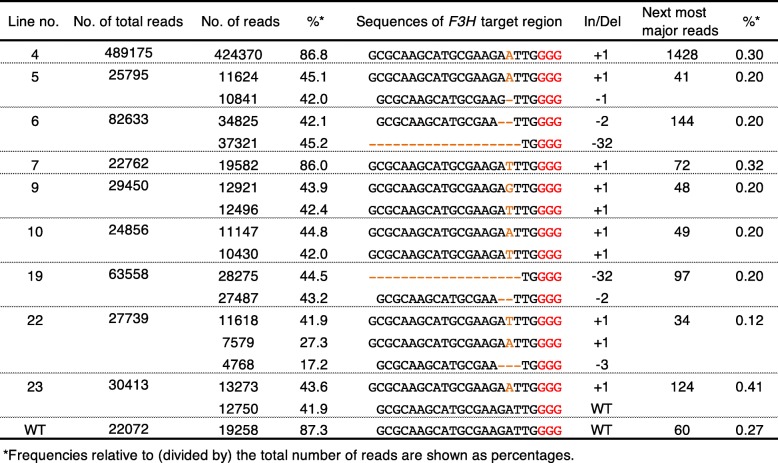
NGS analysis of torenia *F3H* amplicons

### Cultivation in a closed greenhouse and pigment analysis

**Fig. 3:**
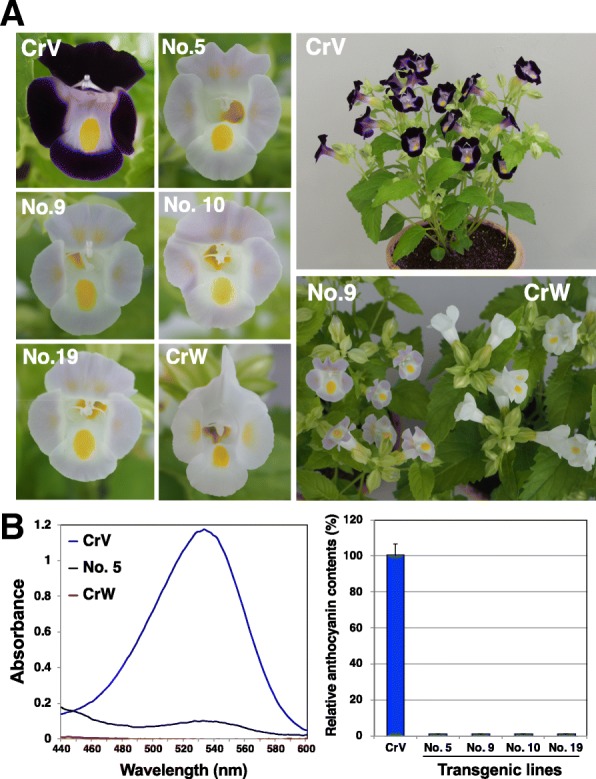
Flowers of transgenic genome-edited torenia plants cultivated in a closed greenhouse and results of pigment analysis. **a** Four biallelic transgenic genome-edited torenia plants were grown in a closed greenhouse. Pictures of typical flowers are shown. CrV and CrW indicate cultivars Crown Violet and Crown White, respectively. CrV was the host plant for transformation, and CrW was a white cultivar for comparison. **b** Pigment analysis by spectrophotometry. Left panel, absorbance spectra of 0.1% HCl–methanol extracts of flower petals (line no. 5, CrV and CrW) are shown in the left panel. Right panel, relative anthocyanin contents of petals of transgenic genome-edited torenia plants and an untransformed control plant (CrV) were determined. Values indicate averages of five flower petals ± standard deviation

To investigate whether the modified flower colors were stable under natural growth conditions, we acclimatized and cultivated the faint blue-flowered transgenic genome-edited lines having mutations in both alleles (nos. 5, 9, 10 and 19) in a closed greenhouse. Their growth was found to be normal, and all tested transgenic genome-edited plants had the same faint blue flowers observed in in vitro flowering (Fig. 3a). The faint blue flowers continued to bloom more than 3 months and the flower color was stable under our cultivation conditions in the greenhouse. Other phenotypes, such as flowering time and plant height, were similar to the WT in all lines. The spectrum absorbance of petal extracts of these four lines displayed a very small peak absorbance at 530 nm, whereas Crown White (a completely white-flowered cultivar) showed none, which indicates the presence of residual anthocyanins in these transgenic genome-edited torenia lines (Fig. 3b). Relative anthocyanin contents were calculated from the absorbance data and are shown in Fig. 3c. These results also indicate that low but detectable levels of anthocyanins were present in the *F3H*-edited plants.

## Discussion

In this study, flower color modification using the CRISPR/Cas9 system was successfully achieved in torenia flowers. Namely, ca. 80% (20/24) of transgenic lines exhibited flower color changes, a sufficiently high efficiency for the practical use of the CRISPR/Cas9 system in this plant species. This value is similar to that achieved by genome editing of the CRISPR/Cas9-targeted torenia *RADIALIS* (*RAD1*) gene controlling floral asymmetry [23]. In that study, 10 out of 12 transgenic lines showed stable phenotypes, but no detailed sequence analyses were performed except for two lines. In regards to flower color modification by genetic transformation in torenia plants, suppression of flavonoid biosynthetic genes has been reported in several studies. For example, inhibition of flower pigmentation was first achieved by the introduction of antisense *CHS* or *DFR* genes, which resulted in flower color changes in 0–89% of transgenic lines, but the degree of color lightening varied among lines [24]. RNAi-targeted *CHS* or *ANS* genes were then applied for flower color suppression, which revealed that RNAi was a more useful method to produce a stable white flower-color phenotype with high efficiency (more than 50%) in torenia [25, 26]. *F3H* genes have also been suppressed by RNAi to produce white flowers in torenia plants, but the efficiency has not been reported [27]. In our study, 20 transgenic T_0_ lines (primary transformants) displayed flower color changes. The edited *F3H* alleles frequently included homozygous and heterozygous biallelic mutations. With respect to the editing sequence pattern, single base insertions (+ 1 bp) were most frequently observed, with deletions such as -1 bp, − 2 bp and -32 bp also occurring in some lines. Su et al. [23] have actually reported the presence of edited *TfRAD1* sequences with a 141-bp deletion or a single-base insertion in two analyzed lines. The pattern of editing may largely depend on the selected target sequences and the type of transformation system (i.e., via callus or direct shoots vs. floral dip). Further analysis is necessary to confirm whether or not CRISPR/Cas9-based genome editing to transform torenia tends to always induce single-base insertions. In contrast, lines no. 23 and 24 had pale violet flowers and harbored a monoallelic mutation (i.e., only one allele was mutated, with the WT sequence remaining). This flower color may be due to the semi-dominant phenotype, in which *F3H* activity is partly functional because of the remaining normal allele. Alternatively, the mutated allele may induce silencing of the normal allele by nonsense-mediated mRNA decay. Line no. 15 actually had different-colored flowers (violet and faint blue) within a single plant, indicating the possibility of chimerism. The presence of three different mutated sequences in line no. 22 also suggests the chimeric nature of this line, although whether this chimerism was derived from an early editing event or occurred during subculture is unknown. Given that line no. 7 set variegated flowers and several lines had a few blue spots, genome editing can probably occur after shoot regeneration and during flowering. Chimerism has also been observed in CRISPR/Cas9 genome-edited flowers of morning glory [14] and rice callus [28]. Genome editing is thus likely ongoing in these transgenic genome-edited torenia lines even though most editing events are considered to occur at the early transformation stage. Further analysis is necessary to gain insights into the chimeric (mosaic) nature of CRISPR/Cas9 system transformation. Because flower color is a visible trait and observation at the single-cell level is possible under the microscope, our materials should be suitable for such studies.

Observation of flowering plants in a closed greenhouse and a pigment analysis uncovered low levels of anthocyanins in *F3H*-mutated petals. This result indicates that torenia can produce low amounts of anthocyanins even when *F3H* is mutated. The reason for this phenomenon is not fully understood, but most likely other endogenous enzymes can catalyze the transformation of flavanones to dihydroflavonols via an unspecific reaction. The *F3H* mutant of Arabidopsis has actually been reported to display a leaky phenotype, with the involvement of flavonol synthase (FLS) and anthocyanidin synthase (ANS), both belonging to the 2-oxoglutarate dependent oxygenase family [29], suspected. In carnation, *F3H* deficiency causes pink flowers in some cultivars [30, 31]. Because further analysis is needed to confirm this hypothesis, we are currently producing *F3H*-edited transgenic tobacco and gentian plants.

Several methods currently exist to assess the outcome of genome editing, such as a PCR/restriction enzyme assay, high-resolution melting (HRM) analysis, and T7 Endonuclease I and TaqMan qPCR assays [32]. Although these methods are useful for screening mutations introduced by the CRISPR/Cas9 system, sequencing analysis provides the most direct evidence to confirm genome editing. Compared with the other methods, however, the cost is problematic. Instead of Sanger sequencing, we analyzed our nine lines of transformants by next-generation amplicon sequencing while indexing multiple samples. The results of NGS were basically consistent with those obtained by Sanger sequencing, thus indicating that NGS and bioinformatic analyses for effectively obtaining edited allele sequence information can save much time and labor. With both methods, however, error incorporation during PCR amplification and NGS must be taken into consideration. For example, sequencing error rates during amplicon sequencing by Illumina Miseq have been well studied [33]. In fact, our NGS results also contained many minor fragments possibly derived from such artificial errors (Table 2 and Additional file 2). In this study, such minor fragments constituted less than 0.5% of fragments at most and did not influence the identification of edited sequences.

As mentioned above, CRISPR/Cas9 is most certainly a valuable tool for flower research using torenia, as editing efficiency is comparably high and the time until flowering is short. The production of effective biallelic mutants enables us to perform gene functional analysis in primary T_0_ plants. We can obtain pale blue-flowered torenia plants by in vitro flowering as early as 4 months after inoculation with *Agrobacterium*. Novel editing tools such as RNA editing and nucleotide substitution by deaminase [17–19] are also promising approaches that we hope can be applied to torenia research in the near future.

## Conclusions

Taken together, our results clearly show that the CRISPR/Cas9 system can efficiently transform flower color in torenia plants. This system will be useful for the functional analysis of genes involved in various traits, such as flower color and shape, flowering time and disease resistance. We hope that various studies on torenia will be performed using the CRISPR/Cas9 system in the future.

## Methods

### Plant materials and transformation

*Torenia fournieri* ‘Crown Violet’ was used as the transformation host, and the cultivar Crown White was also used as a control for pigment analysis. Both are clonal lines provided by Dr. Ryutaro Aida (National Institute of Floricultural Science, Japan) and also used in our previous study [21]. Plants maintained by in vitro culture were used for transformation as described previously [21]. Briefly, leaf sections were excised and co-cultivated with *Agrobacterium* harboring a pSKAN-pcoCas9-TfF3H vector (Fig. 1a) for 1 week. Kanamycin-resistant calli were selected, and regenerated shoots were transferred to rooting medium. After rooting, transgenic plants were cultured in vitro until flowering, and flower color was observed. After acclimatization, some transgenic genome-edited plants were grown in a closed greenhouse and cultivated until flowering.

### Construction of a binary vector for genome editing

The binary CRIPSR/Cas9 vector, pSKAN-pcoCas9-TfF3H (Fig. 1), was constructed and used for plant transformation. Briefly, pSKAN-pcoCas9 was first constructed by replacing the *uidA* (*gus*) gene of pSKAN35SGUS [34] by the plant codon-optimized SpCas9 (pcoCas9) [35] purchased from Addgene (Cambridge, MA, USA) and an Arabidopsis HSP18.2 terminator [36]. A synthesized single-guide RNA targeting the torenia *F3H* gene was then introduced to pSKAN-pcoCas by restriction enzyme treatment and ligation, resulting in pSKAN-pcoCas9-TfF3H (Fig. 1a). Target site was selected manually in the *F3H* coding region and located about 200 bp downstream from translation start codon (Fig. 1b). The vector was transformed into *Agrobacterium tumefaciens* strain EHA101 by electroporation.

### Anthocyanin analysis

Flowers were collected from greenhouse-grown plants, and their petals were extracted with methanol containing 0.1% HCl. Anthocyanin contents were determined by measurement of OD at 530 nm spectrophotometrically.

### Sanger sequencing analysis

Leaf extracts of transgenic plant lines were subjected to PCR using primer pairs TfF3HU7 (5*′*-AGCAGGACCACTAACCCTAACTTC-3*′*) and TfF3HL371 (5*′*- GGTGACTCGAAACAATGAAACCTC-3*′*) (Fig. 1b) with MightyAmp DNA polymerase (Takara Bio., Shiga, Japan) according to the manufacturer’s instruction. After purification, the amplified fragments were directly subjected to Sanger sequencing analysis using a BigDye terminator ver. 1.1 cycle sequencing kit and the forward TfF3HU7 primer on an ABI PRISM 3130xl DNA sequencer (Applied Biosystems, Foster City, CA, USA). The sequence chromatograms were analyzed manually or using the DSDecode program [37]. Amplified fragments of some selected lines were also subcloned into PCR4-TOPO (Invitrogen, Carlsbad, CA) and sequenced with M13 M4 or M13RV primers as described above.

### NGS analysis

Genomic DNAs were isolated from leaf samples of in vitro grown transgenic torenia plants using the GeneElute Genome DNA Isolation system (Sigma-Aldrich, St Louis, MO, USA) in accordance with the manufacturer’s instructions. PCR amplification was performed using a KAPA HiFi HotStart ReadyMixPCR kit (Kapa Biosystems, Wilmington, MA, USA). Primers TfF3H1U7_57mer and TfF3H1L371_57mer (Additional file 1: Table S1) were used for the first PCR round, which was carried out under the following conditions: 3 min at 95 °C, followed by 20 cycles of 30 s at 95 °C, 30 s at 55 °C and 30 s at 72 °C, and then 5 min at 72 °C. Conditions for the second PCR round were as follows: 3 min at 95 °C, followed by 12 cycles of 30 s at 95 °C, 30 s at 55 °C and 30 s at 72 °C, and a final step of 5 min at 72 °C. The second PCR was performed using D50x and D70x primers (Additional file 1: Table S1) for indexing. Products from the two PCR rounds were purified using AMPure XP beads (Beckman Coulter, High Wycombe, UK). Amplification of the second set of PCR products was checked on a fragment analyzer (Advanced Analytical Technologies, Ankeny, IA, USA) using a High Sensitivity NGS Fragment Analysis kit. All PCR products were mixed at the same volume ratio. Concentrations of mixed PCR products, namely, a bulked library, were measured by qPCR using a KAPA Library Quantification kit. The final concentration of the bulked library was diluted to 6 pM. As a control, PhiX (Illumina control library version 2; Illumina, San Diego, USA) was spiked in the bulked library at 50% (*v*/v). Sequencing was performed on an Illumina MiSeq system using a MiSeq Reagent v2 kit (500 cycles). The resulting raw sequence reads were preprocessed using the FASTX toolkit as follows. First, reads shorter than 40 bases were discarded, and the remaining reads were trimmed at 230 bases. Second, quality filtering of the remaining 40–230-base reads was performed using q20 and p80 parameters. Third, unpaired reads were discarded. Fourth, read pairs were merged into a single contiguous sequence (fragment) using a fastq-join script [38]. Finally, unique fragments were counted.

## Additional files

Additional file 1: **Figure S1.** Schematic representation of the flavonoid biosynthesis pathway in torenia. ANS, anthocyanidin synthase; C4H, cinnamate 4-hydroxylase; CHI, chalcone isomerase; CHS, chalcone synthase; 4CL, 4-coumarate: CoA ligase; DFR, dihydroflavonol 4-reductase; DHK, dihydrokaempferol; DHM, dihydromyricetin; DHQ, dihydroquercetin; F3H, flavanone 3-hydroxylase (target gene in this study); F3*′*H, flavonoid 3*′* -hydroxylase; F3*′*,5*′*H, flavonoid 3*′*, 5*′* -hydroxylase; FNSII, flavone synthase II; GT, glucosyltransferase; MT, methyltransferase; PAL, phenylalanine ammonia lyase. **Figure S2.** Sequence chromatograms of the *TfF3H* gene in different transgenic torenia lines. PCR products were subjected to Sanger sequencing using the TfF3HU7 primer. The region of the sequence chromatogram including the target site is enlarged. Transgenic line numbers are shown on each chromatogram. WT indicates the wild-type sequence (cv. Crown Violet). **Table S1.** Primers used for multiplex amplicon sequencing by NGS. (PDF 1803 kb)

Additional file 2: The results of next-generation amplicon sequencing of cv. Crown Violet and nine genome-edited torenia lines. Numbers after sequence names in the Excel worksheets are sequential serial numbers and read counts of each fragment. (XLSX 668 kb)

## Abbreviations

CRISPR: Clustered regularly interspaced short palindromic repeats
F3H: Flavanone 3-hydroxylase
NGS: Next-generation sequencing
PCR: Polymerase chain reaction
