# Supplementary figures and images for: Application of the CRISPR/Cas9 system for modification of flower color in Torenia fournieri
Source: BMC Plant Biol. 2018 Dec 5;18:331. doi: 10.1186/s12870-018-1539-3 (PMC6280492; doi:10.1186/s12870-018-1539-3)

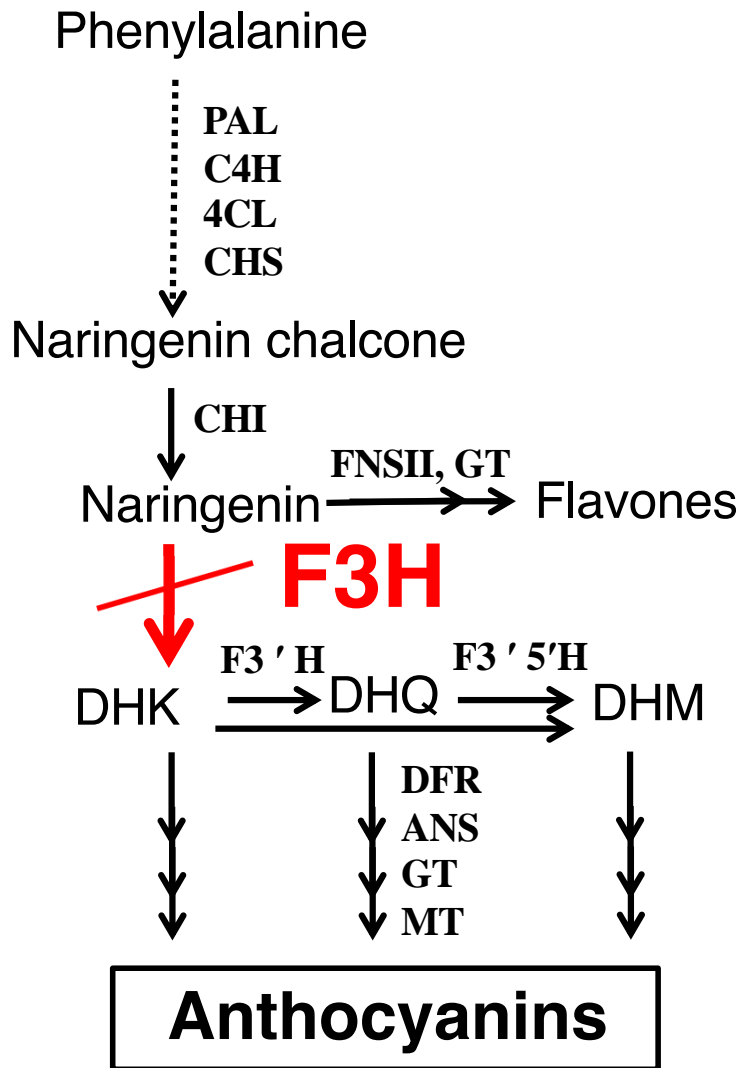

Supplement: Supplementary file 1 — Figure S1. Schematic representation of the flavonoid biosynthesis pathway in torenia. ANS, anthocyanidin synthase; C4H, cinnamate 4-hydroxylase; CHI, chalcone isomerase; CHS, chalcone synthase; 4CL, 4-coumarate: CoA ligase; DFR, dihydroflavonol 4-reductase; DHK, dihydrokaempferol; DHM, dihydromyricetin; DHQ, dihydroquercetin; F3H, flavanone 3-hydroxylase (target gene in this study); F3′H, flavonoid 3′ -hydroxylase; F3′,5′H, flavonoid 3′, 5′ -hydroxylase; FNSII, flavone synthase II; GT, glucosyltransferase; MT, methyltransferase; PAL, phenylalanine ammonia lyase. Figure S2. Sequence chromatograms of the TfF3H gene in different transgenic torenia lines. PCR products were subjected to Sanger sequencing using the TfF3HU7 primer. The region of the sequence chromatogram including the target site is enlarged. Transgenic line numbers are shown on each chromatogram. WT indicates the wild-type sequence (cv. Crown Violet). Table S1. Primers used for multiplex amplicon sequencing by NGS. (PDF 1803 kb) [file 12870_2018_1539_MOESM1_ESM.pdf]
